# An in vitro comparative evaluation of surface roughness characteristics of different orthodontic archwires: An atomic force microscopy study

**DOI:** 10.34172/joddd.2022.015

**Published:** 2022-10-15

**Authors:** Reshma Mohan, Ravindra Kumar Jain

**Affiliations:** Department of Orthodontics and Dentofacial Orthopaedics, Saveetha Dental College and Hospital, Saveetha Institute of Medical and Technical Sciences, Saveetha University, Tamil Nadu, India

**Keywords:** Atomic force microscopy, Orthodontic archwire, SmartArch wire, Surface roughness, Surface treatment

## Abstract

**Background.** The present study evaluated and compared the surface roughness (SR) of five different types of orthodontic archwires made by two different manufacturers.

**Methods.** In this in vitro study, 10 samples of five different archwires comprising of three types of shape memory wires, SmartArch (Ormco), Damon (Ormco), Heat-activated NiTi (HANT) (G&H Orthodontics), Stainless Steel wire (SS) (Ormco), and conventional NiTi (G&H Orthodontics) were examined by atomic force microscopy (AFM). The processing of 3D images was carried out using Gwyddion software, from which the root mean square (rms), the roughness average (Ra), and the maximum height (mh) of the scanned surface profile were documented. The data were analyzed with one-way ANOVA followed by post hoc Tukey tests for intergroup comparisons.

**Results.** The mean SR of SS wires was the least (Ra=8.70±0.17), followed by NiTi wires (10.29±2.00) with a significant difference between them (*P*<0.05). Among the three shape-memory wires, the HANT wires had the least SR (Ra=22.97±16.56) compared to SmartArch wires (Ra=25.55±3.78) and Damon wires (Ra=25.67±4.54), but the difference was not significant (*P*>0.05).

**Conclusion.** The SS wires by Ormco had the least SR followed by G&H orthodontics NiTi wires. The three different shape-memory wires tested had no significant difference in SR values.

## Introduction

 Several properties explored in the search for an ideal archwire in orthodontics include biostability, friction, formability, weldability, resilience, and spring-back.^1^ Of late, a variety of alloys for orthodontic archwires have been introduced due to the groundbreaking research work carried out in orthodontic materials. The orthodontist can now choose an ideal wire for the necessary clinical condition. The surface roughness (SR) of archwires determines the surface area in contact and can affect the frictional characteristics, corrosion behavior, and biocompatibility.^2^ Studieshave shown that all these properties influence the clinical performance of orthodontic archwires.^3^ Furthermore, surface topography can critically alter the frictional characteristics and corrosion resistance, affecting the efficiency of archwires.^4^ SR can also influence plaque accumulation and this, in turn, has an essential role in other properties previously described.^3^ Archwires used for orthodontic treatment are subjected to various mechanical, chemical, and thermal stresses in the patient’s mouth and the surface topography of the wire can influence wires.

 SR analysis of different archwires is important for evaluating archwire performance. Previously, SR evaluation was performed with surface profilometry,^2^ where a thin tip is employed in a single line of a selected area to scan the surface topography. The disadvantage of this method was that measuring surface defects near the scan line was difficult. Also, profilometry is a more invasive technique that could deteriorate the surface while scanning. The demand for non-invasive techniques leads to the introduction of new analysis methods, such as optical methods^5^ and the scanning tunneling microscope method.^6^ With these techniques, it became possible to perform a surface analysis on preselected areas of archwires without directly interacting with them. Scanning probe microscopy involves the following types: the atomic force microscope (AFM),^7^ scanning tunneling microscopes, and the magnetic force microscope. As the AFM can provide 3D images of surface morphology with high resolution, it is considered the most suitable tool for quantifying surface topography.^2,8,9^ It belongs to a class of tools that use a scanning probe that uses the interatomic interactions to acquire information on the detected surfaces using sensors.^8,10,11^ These sensors consist of sharp points that interact with the specimen surface. In addition to obtaining 3D images in the AFM technique, 2D images are also prepared simultaneously, and the samples can be re-assessed without being destroyed.^2^ The main drawback of AFM is the small scan size and slow scanning speed, which often impedes the complete evaluation of the sample.

 This study aimed to measure and compare the surface topography of five different archwires by two different manufacturers using AFM.

## Materials and Methods

 Five different rectangular cross-sections of orthodontic archwires were considered for this study: SmartArch wires (Ormco), Damon wires (Ormco), HANT wires (G&H Orthodontics), NiTi wires (G&H Orthodontics), and SS wires (Ormco). Ten samples from each wire group were used. From the ten samples, 5 mm of wire was cut from the end of the archwires. These were viewed under the AFM under ambient conditions.

 The samples were tested using atomic force microscopy at the Department of Metallurgical and Materials Engineering, Indian Institute of Technology Madras, Chennai. The Department is equipped with a Digital Instruments Dimension^TM^ 3100 Atomic Force Microscope, which is used for surface profiling with high resolution at the Angstrom level.

 A metal holder with cyanoacrylate glue was used to attach the samples. Five areas of the archwire surface were randomly selected and assessed for each sample. The Gwyddion software was used to process the 3D images. The average surface roughness (Ra), the root mean square (rms), and the maximum height (mh) were the parameters assessed by the software. The arithmetic mean of absolute values and root mean square of the scanned surface were represented by Ra and rms, respectively, whereas mh represented the maximum height of the surface profile peak.

 Statistical tests were performed using SPSS 23.0.0. Descriptive statistics were used along with one-way ANOVA, followed by post hoc Tukey tests, to compare the surface characteristics of the different orthodontic archwires.

## Results

**Figure 1:**
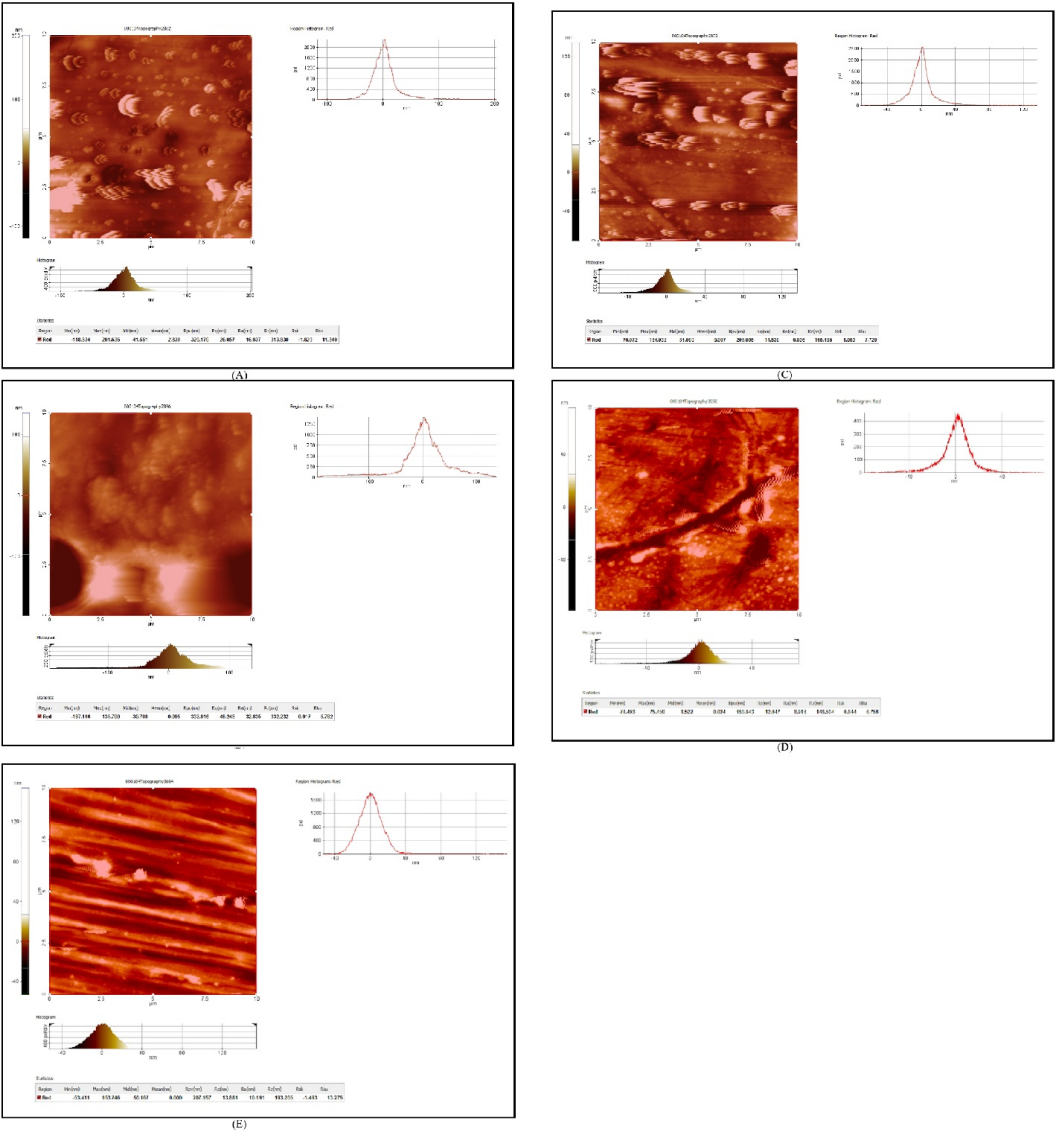
Representative three-dimensional AFM topography images of the eight samples of orthodontic archwires: (A) SmartArch wire, (B) Damon wire, (C) Heat-Activated NiTi, (D) Stainless Steel wire, and (E) NiTi wire.

 In all the wires tested, many topographic irregularities were reported. The 3-D AFM topography images of all the tested wires were assessed (Figure 1). The two-dimensional images were also analyzed to evaluate the nanodomain dimensions. Table 1 depicts the means and standard deviations of the three roughness parameters used to analyze the surface topography of each archwire quantitatively.

**Table 1:** Means and standard deviations of the surface roughness average (Ra), the root mean square (rms), and the maximum height (mh)

**Archwires**	**Ra, Mean±SD**	**rms, Mean±S.D**	**mh, Mean±SD**
HANT wire	22.97 ± 16.55	20.31 ± 6.38	286.68 ± 64.57
Damon Q wire	25.67 ± 4.54	34.31 ± 8.72	258.16 ± 63.39
SmartArch wire	25.55 ± 3.78	32.25 ± 5.04	366.53 ± 89.41
Stainless steel wire	8.69 ± 0.18	14.49 ± 1.61	209.41 ± 57.42
NiTi wire	10.29 ± 2.12	15.99 ± 3.50	171.16 ± 35.73

Table 2 presents the results of ANOVA for statistical comparison of differences in surface topography parameters between the different archwires. Among the five archwires, the least SR was reported in SS wires (Ra = 8.69 ± 0.18), followed by conventional NiTi wires (Ra = 10.29 ± 2.12), HANT wires (Ra = 22.97 ± 16.55), SmartArch wires (Ra = 25.55 ± 3.78), and Damon wires (Ra = 25.67 ± 4.54), with significant differences between them (*P* < 0.05).

**Table 2:** *P* values from statistical analysis of archwire roughness parameters (ANOVA with post hoc Tukey tests)

**Tukey multiple comparison tests**	**Ra**	**rms**	**mh**
HANT wire vs. Damon wires	ns	***	ns
HANT wires vs. SmartArch wires	ns	***	ns
HANT wires vs. NiTi wires	**	ns	*
HANT wires vs. Stainless steel wires	**	ns	ns
Damon wires vs. SmartArch wires	ns	ns	**
Damon Wires vs. NiTi wires	***	***	*
Damon wires vs. Stainless steel wires	***	***	ns
SmartArch wires vs. NiTi wires	***	***	***
SmartArch wires vs. Stainless steel wires	***	***	***
NiTi wires vs. Stainless Steel wires	ns	ns	ns

ns indicates not significant. *P < 0.05; **P < 0.01; and ***P < 0.001 indicate statistically significant differences between the two archwires.

 Among the NiTi wires, conventional NiTi wire exhibited the least SR, and the difference in SR was significant between them (*P* < 0.05). Among the shape memory wires, the HANT wires exhibited the least SR, but the difference was not significant (*P* > 0.05).

## Discussion

 In the present study, SS wires exhibited the least SR compared to NiTi and shape-memory wires. Among the CuNiTi archwires, the heat-activated NiTi exhibited the least roughness characteristics. However, this was not statistically significant. Various studies have compared the SR of NiTi and NiTi-coated wires and TMA and TMA-coated wires, etc. However, fewer studies have compared the surface topography of shape memory wires with NiTi and SS wires.

 Stainless steel exhibited the least SR in the present study. D’Antò et al^8^ compared four nickel-titanium wires, three beta-titanium wires, and one stainless steel wire using AFM technology. Yousif and Abd El-Karim^12^ compared stainless steel (SS), titanium molybdenum alloy (TMA), nitinol (NiTi), and copper NiTi wires. Both studies demonstrated that stainless steel wires had the lowest SR, frictional coefficient, and sliding resistance. Bourauel et al^2^ compared 11 NiTi wires, and Prososki et al^13^ compared 9 NiTi wires with one SS wire and one beta-titanium wire. The authors in both studies noted that the SS wire had the smoothest surface. Additionally, Shin et al^14^ compared NiTi and SS wires and reported that SS wires were smoother. Amini et al^15^ also observed similar results, but this was not statistically significant. However, they also reported that the NiTi wire of American Orthodontics was smoother than the SS wire, which could be attributed to random sampling methods. Also, the selected parts may have been compromised during manufacturing processes or delivery.

 In the current study, the surface topographic characteristics of archwires were analyzed using AFM. AFM is a non-invasive method compared to surface profilometry and an established method; hence, we used it in our study. Yousif and Abd El-Karim^12^ studied the comparison of SR of orthodontic archwires obtained using AFM and digital optical microscopy, reporting no significant difference between the values obtained from these methods, indicating that both methods are effective in determining the SR of archwires.

 According to the Amontons-Coulomb Law (the first law of friction), the frictional coefficient of an archwire depends on the wire roughness and its physical characteristics.^16^ The frictional force between brackets and wires is an unfavorable factor that can influence the tooth movement during sliding mechanics. Several studies^17,18^ reported a correlation between SR and friction. However, the orthodontic movement of teeth is an intricate process associated with several important factors. The production technique is one of the important factors affecting archwires’ surface topography.^8^ It has been observed that the SR of a group of wires produced by the same manufacturer is almost the same in the given wires. Other factors that could influence the surface characteristics of a wire can be the materials used, the coatings used, and the manufacturing technique.^19,20^

## Conclusion

 Within the limitations of the study, it was found that the surface quality of stainless steel wire was less rough compared to the other wires. All the tested shape-memory wires exhibited similar SR. Apart from influencing the effectiveness of sliding mechanics, the SR could also affect the corrosion nature and the appeal of orthodontic components. Hence, the surface quality of the orthodontic archwires should be enhanced during the manufacturing process.

## Acknowledgments

 We would like to express our sincere gratitude to our research supervisor Dr. Ravindra Kumar Jain, associate professor, Department of Orthodontics, Saveetha Dental College and Hospitals, Chennai, for his guidance and encouragement with our work in all stages. We would also like to acknowledge Dr. Chirag Chawan for his contribution to this study.

## Authors’ Contributions

 Both authors have equally contributed to the design, conception, and preparation of this research.

## Funding

 No funding.

## Ethics Approval

 This study was approved by the Scientific Review Board of Saveetha Dental College with the ethical clearance number IHEC/SDC/ORTHO-1903/21/171.

## Competing Interests

 No conflicts of interest.
